# Molecular Basis and Role of Siglec-7 Ligand Expression on Chronic Lymphocytic Leukemia B Cells

**DOI:** 10.3389/fimmu.2022.840388

**Published:** 2022-05-31

**Authors:** Lan-Yi Chang, Suh-Yuen Liang, Shao-Chia Lu, Huan Chuan Tseng, Ho-Yang Tsai, Chin-Ju Tang, Marcelia Sugata, Yi-Ju Chen, Yu-Ju Chen, Shang-Ju Wu, Kuo-I Lin, Kay-Hooi Khoo, Takashi Angata

**Affiliations:** ^1^ Institute of Biological Chemistry, Academia Sinica, Taipei, Taiwan; ^2^ Genomics Research Center, Academia Sinica, Taipei, Taiwan; ^3^ Institute of Biochemical Sciences, National Taiwan University, Taipei, Taiwan; ^4^ Institute of Chemistry, Academia Sinica, Taipei, Taiwan; ^5^ Division of Hematology, Department of Internal Medicine, National Taiwan University Hospital, Taipei, Taiwan

**Keywords:** chronic lymphocytic leukemia, natural killer cells, Siglec-7, sialomucin, ST6GalNAc-IV, Core 2 GlcNAc transferase

## Abstract

Siglec-7 (sialic acid–binding immunoglobulin-like lectin 7) is an immune checkpoint-like glycan recognition protein on natural killer (NK) cells. Cancer cells often upregulate Siglec ligands to subvert immunosurveillance, but the molecular basis of Siglec ligands has been elusive. In this study, we investigated Siglec-7 ligands on chronic lymphocytic leukemia (CLL) B cells. CLL B cells express higher levels of Siglec-7 ligands compared with healthy donor B cells, and enzymatic removal of sialic acids or sialomucins makes them more sensitive to NK cell cytotoxicity. Gene knockout experiments have revealed that the sialyltransferase ST6GalNAc-IV is responsible for the biosynthesis of disialyl-T (Neu5Acα2–3Galβ1–3[Neu5Acα2–6]GalNAcα1–), which is the glycotope recognized by Siglec-7, and that CD162 and CD45 are the major carriers of this glycotope on CLL B cells. Analysis of public transcriptomic datasets indicated that the low expression of *GCNT1* (encoding core 2 GlcNAc transferase, an enzyme that competes against ST6GalNAc-IV) and high expression of *ST6GALNAC4* (encoding ST6GalNAc-IV) in CLL B cells, together enhancing the expression of the disialyl-T glycotope, are associated with poor patient prognosis. Taken together, our results determined the molecular basis of Siglec-7 ligand overexpression that protects CLL B cells from NK cell cytotoxicity and identified disialyl-T as a potential prognostic marker of CLL.

## Introduction

Chronic lymphocytic leukemia (CLL) is the most common type of hematopoietic malignancy (1, 2). CLL develops over a long period of time by the accumulation of mature clonal B lymphocytes that proliferate in an uncontrolled manner and/or fail to undergo cell death. Clinical outcome of CLL is influenced by many factors, and the mutation status of immunoglobulin heavy chain variable region (*IGHV*), reflecting the differentiation stage of the B cell clone that eventually gives rise to CLL, is a strong prognostic factor (3, 4). Survival of CLL cells depends on the signaling through B-cell receptor, which may recognize autoantigen or environmental antigen (5–7). Approval of drugs targeting the B-cell receptor signaling pathway (i.e., Btk and PI3Kδ inhibitors) and the anti-apoptotic protein Bcl2 inhibitor has revolutionized the treatment of CLL in the past decade (8). However, drug resistance eventually develops in many patients, necessitating new therapeutic approaches. Recent success in clinical trials of chimeric antigen receptor–transduced T cell and NK cell therapies has marked the beginning of a new era in CLL therapy (9, 10). The identification of factors influencing the success of cell-based CLL therapy is thus of clinical interest.

NK cells are equipped with various germline-encoded receptor proteins working as environmental sensors, and the sum of the inputs from activating and inhibitory receptors determines the cellular response (11–13). A previous study found that genetic polymorphisms determining the ratio between inhibitory and activating killer immunoglobulin-like receptors are associated with susceptibility to CLL (14), suggesting the importance of NK cell–mediated immunosurveillance in CLL. Siglec-7 (sialic acid–binding immunoglobulin-like lectin 7), also known as p75/AIRM-1, is one of the inhibitory receptors on NK cells (15, 16) and is considered to be a potential cancer immunotherapy target (17, 18). Many Siglecs, from a family of glycan recognition proteins expressed on various leukocytes, have immune checkpoint-like properties and contribute to the fine-tuning of immune responses (19, 20). Each Siglec shows a unique expression pattern and its own glycan recognition preference (21, 22). Research has shown that neutralization of Siglec-7 (expressed primarily on NK cells) and Siglec-9 (expressed primarily on myeloid cells but also on cytotoxic T cells in cancer patients) with an antibody can modulate the responses of killer lymphocytes in favor of cancer elimination (18, 23). Removal of sialic acid, a sugar residue recognized by Siglecs, from cancer cells also sensitizes them to cellular cytotoxicity by killer lymphocytes and other mechanisms (17, 24–26).

These previous studies demonstrated that the sialic acid–Siglec axis is a promising target for checkpoint inhibitor–type intervention in cancer treatment. However, our knowledge regarding the identity of Siglec ligands on cancer cells, consisting of the glycan epitope (*glycotope*) recognized by Siglec and the glycoproteins (*counterreceptors*) that exhibit the glycotope, is still limited (27, 28). The inherent difficulties in deciphering glycan-based recognition events include the low affinity of interaction between the glycan recognition protein and cognate glycotope (with the Kd value often being in the order of 10^-3^ M), complexity of glycan structures and biosynthesis pathways, redundancy in counterreceptors (i.e., the same glycotope can be exhibited in multiple glycoproteins), and the membrane-associated nature of functional ligands, among others. Regardless, understanding the molecular basis of Siglec-based immune subversion by cancer is crucial to improving the efficacy of cancer therapy. In this study, we used a combination of approaches to determine the molecular basis of Siglec-7 ligands on CLL B cells and further identified a potential prognostic marker of CLL *via* bioinformatic analysis of public transcriptomic datasets.

## Materials and Methods

### Collection of Donor Blood and Purification of B Cells

The institutional review boards of the National Taiwan University Hospital and Academia Sinica approved this study (approval nos. 201907037RINA and AS-IRB-BM-19043, respectively). Taiwanese CLL patients were recruited at the National Taiwan University Hospital. Informed consent was obtained from each participant before peripheral blood samples were collected. The characteristics of the patients are summarized in **Supplementary Table 1**. Blood samples from healthy donors were obtained from the Taipei Blood Center (Taipei, Taiwan). B cells were purified from the blood samples by density gradient centrifugation using Ficoll-Paque PLUS (cat. no. 17-1440-03; Cytiva, Marlborough, MA, USA) followed by affinity purification with CD19 MicroBeads (cat. no. 130-050-301; Miltenyi Biotec, Bergisch Gladbach, Germany), as previously described (29).

### Cell Lines

The human CLL cell lines JVM-3, MEC-1, and MEC-2 were obtained from DSMZ–German Collection of Microorganisms and Cell Cultures (Braunschweig, Germany). JVM-3 was maintained in RPMI-1640 medium containing 10% fetal bovine serum (FBS) and 1% penicillin/streptomycin (Pen/Strep; Thermo Fisher Scientific, Waltham, MA, USA), whereas MEC-1 and MEC-2 were maintained in IMDM containing 10% FBS and 1% Pen/Strep. The human NK cell line NK-92MI (30) was obtained from Bioresources Collection and Research Center (Hsinchu, Taiwan) and maintained in MEMα containing 12.5% horse serum, 12.5% FBS, 1% Pen/Strep, 0.2 mM inositol, 0.1 mM 2-mercaptoethanol, and 0.02 mM folic acid.

### Antibodies and Other Reagents

Allophycocyanin-labeled anti-CD43 (clone L10) was obtained from Thermo Fisher Scientific. Phycoerythrin (PE)-labeled anti-CD43 (clone CD43-10G7), PE-labeled anti-CD45 (clone KPL1), and PE-labeled anti-CD162/PSGL-1 (clone 2D1) were purchased from Biolegend (San Diego, CA, USA). Recombinant Siglec–Fcs (consisting of an extracellular lectin domain of Siglec and human immunoglobulin G1 hinge–Fc region, with a FLAG tag in between) were prepared in-house (31). Fluorescein– and Alexa Fluor 647–labeled anti-human immunoglobulin G antibodies were acquired from Jackson ImmunoResearch (West Grove, PA, USA).

Sialidase (neuraminidase) from *Arthrobacter ureafaciens* was purchased from Nacalai (Kyoto, Japan). O-sialoglycoprotein endopeptidase (OSGP-EP) was acquired from Cedarlane Laboratories (Burlington, Ontario, Canada). Benzyl-2-acetamido-2-deoxy-α-d-galactopyranoside (benzyl-α-GalNAc) and kifunensine were obtained from Millipore Sigma (St. Louis, MO, USA). dl-Threo-1-phenyl-2-decanoylamino-3-morpholino-1-propanol was purchased from Cayman Chemical (Ann Arbor, MI, USA).

### Proximity Labeling of JVM-3 Cells With Siglec-7–Fc and Identification of Counterreceptor Candidates

Identification of Siglec-7 counterreceptors was attempted with proximity labeling as previously described (32). In brief, JVM-3 cells (1×10^7^) were incubated with Siglec-7–Fc (10 μg) or binding-deficient mutant Siglec-7(R124A)–Fc (10 μg) precomplexed with peroxidase-conjugated anti-FLAG antibody (5 μg; cat. no. A8592; Millipore Sigma), followed by incubation with biotin labeling reagent (10 μM biotin tyramide and 10 mM H_2_O_2_ in Tris-buffered saline). Biotinylated proteins were purified from cell lysates with Dynabeads MyOne Streptavidin C1 (Thermo Fisher Scientific), eluted by heat denaturation in sample buffer (Bio-Rad, Hercules, CA, USA), and subjected to sodium dodecyl sulfate–polyacrylamide gel electrophoresis and in-gel trypsin digestion. The peptides were analyzed by liquid chromatography with tandem mass spectrometry (LC–MS/MS) using an Orbitrap Elite hybrid mass spectrometer (Thermo Fisher Scientific). The raw data were processed using Proteome Discoverer 2.1 (Thermo Fisher Scientific), and peptide identification was performed using Mascot (version 2.3.2) and SEQUEST against the Swiss-Prot human database with a strict false discovery rate of 0.01. Label-free quantification was performed using the peak area of each precursor ion with a mass precision of 2 ppm. Details of the analysis are described in **Supplementary Materials and Methods**. The proteomics data set was deposited to ProteomeXchange *via* the PRIDE database (accession no. PXD024690).

### Gene Expression Analysis With Quantitative Real-Time Polymerase Chain Reaction

The transcript levels of the genes of interest were analyzed with quantitative real-time polymerase chain reaction (qRT-PCR) using commercial primer–probe sets (TaqMan Real-Time PCR Assay; Thermo Fisher Scientific; **Supplementary Table 2**), in accordance with the protocols provided by the manufacturer. First-strand complementary DNA was prepared from 1 μg of total RNA extracted from the cells using a SuperScript III First-Strand Synthesis System with random hexamer primers (Thermo Fisher Scientific). The preparation was then used for the qRT-PCR assays with a FastStart Universal Probe Master (Roche, Mannheim, Germany) in a StepOnePlus Real-Time PCR System (Thermo Fisher Scientific).

### Expression of Siglec-7 in NK-92MI Cells

NK-92MI does not express Siglec-7 (33). We thus expressed Siglec-7 by lentiviral transduction as previously described (34). Siglec-7+ cells (NK-92MI/S7) were sorted by fluorescence-activated cell sorting twice. They were later used without further cloning.

### Preparation of Gene-Edited JVM-3 and MEC-1 Cells With CRISPR–Cas9

To obtain JVM-3 and MEC-1 sublines lacking the genes of interest, we introduced *Streptococcus pyogenes* Cas9 and single-guide RNA (sgRNA) expression constructs *via* lentiviral transduction. Lentiviruses for the expression of Cas9 (p5w.Cas9.Pbsd) and sgRNAs (pU6-gRNA.Ppuro) were obtained from RNA Technology Platform and Gene Manipulation Core (National Biotechnology Research Park and Academia Sinica, Taipei, Taiwan). Transduced cells were subjected to drug selection and further sorted to select the population that lost the target protein (as revealed by antibody staining) or the target glycotope (as revealed by lectin or antibody staining; **Supplementary Figure 1**). Sorted cells were propagated, and the indels in the target gene were analyzed by genomic PCR and DNA fragment length analysis (with 3730xl DNA Analyzer and GeneMapper Software v4.0, Applied Biosystems/Thermo Fisher Scientific; outsourced to Genomics, New Taipei City, Taiwan; **Supplementary Figure 1**). Sorted cells were used without further cloning. Owing to the pseudo-tetraploid nature of the JVM-3 cell line, sequencing-based genotyping was not conducted. The sequences of the sgRNA and PCR primers used for DNA fragment length analysis are summarized in **Supplementary Table 3**.

### NK Cell Cytotoxicity Assay

Target cells were labeled with 5 μM calcein acetoxymethyl ester (Thermo Fisher Scientific) in Dulbecco’s PBS, washed three times with 5% FBS in Dulbecco’s PBS, and mixed with NK-92MI/S7 at an effector/target ratio in the range of 1:1 to 10:1 in 96-well conical bottom plates (cat. no. 249935, Thermo Fisher Scientific). After a 4-h incubation at 37°C in a CO_2_ incubator, the plate was centrifuged (at 600 g, 3 min), the supernatant (150 μL) was transferred to a fresh chimney plate (cat. no. 655096, Greiner Bio-One; Kremsmünster, Austria), and fluorescence intensity (excitation: 485 nm; emission: 535 nm) was measured with a plate reader (SpectraMax Paradigm; Molecular Devices, San Jose, CA, USA). Specific lysis was calculated with the following formula:


$$SpecificLysis\left(\%\right)=100\times \left(F_{E+T}-F_{T}\right)/\left(F_{max}-F_{T}\right)$$

where F_E+T_, F_T_, and F_max_ represent fluorescence in the supernatant from the effector + target, target alone, and maximum release by detergent lysis, respectively.

### Quantitative Analysis of O-Glycans With LC–MS/MS

O-glycans were released from cells by alkaline reductive elimination, permethylated, and subjected to reversed-phase C18 nanoLC–MS/MS analysis as previously described (35). The major O-glycans detected and verified by MS/MS were relatively quantified by the peak areas of their extracted ion chromatograms. Details of the analysis are described in **Supplementary Materials and Methods**.

### International Cancer Genome Consortium CLL Transcriptomic Data Analysis

Access to the data sets for CLL patients was granted by the Data Access Compliance Office of the International Cancer Genome Consortium (DACO-1071633). RNA sequencing–based transcriptomic data sets for CLL patients (EGAD00001000258 and EGAD00001001443) were downloaded and analyzed using a Taiwania 1 supercomputer at the National High-Performance Computing Center (Hsinchu, Taiwan) and GNU Parallel (36). RNA sequencing data of the patients with CLL or small cell lymphoma and with survival status (n = 255 and n = 9, respectively; total n = 264) were included in the analysis. Patient data was obtained as metadata from the International Cancer Genome Consortium, and supplemented with *IGHV* mutation status from (37). Details of the analysis are described in **Supplementary Materials and Methods**.

### Statistics

**Figure 1:**
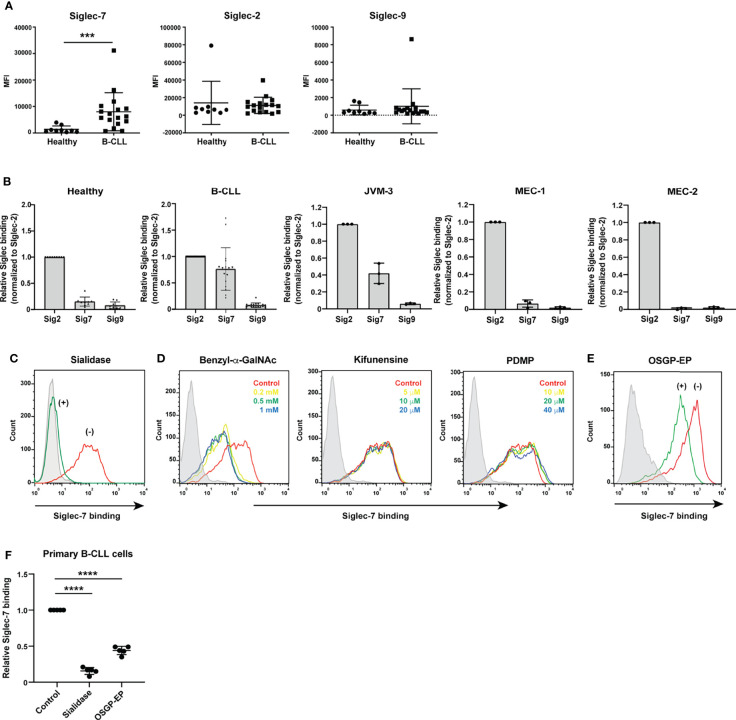
Siglec-7 ligands are highly expressed on B cells from patients with chronic lymphocytic leukemia (CLL). **(A)** Comparison of Siglec ligand levels between B cells from healthy donors and those from CLL patients. The difference in Siglec-7–Fc binding (expressed as median fluorescence intensity [MFI]) to B cells between healthy donors (n = 9) and CLL patients (n = 17) was statistically significant (***P < 0.001, Mann–Whitney test), whereas the difference in CD22/Siglec-2–Fc and Siglec-9–Fc binding between the two groups was not (*P* = 0.33 and 0.38, respectively; Mann–Whitney test). Bars represent mean ± SD. **(B)** Siglec ligands in primary B cells and CLL B cell lines. JVM-3, MEC-1, and MEC-2 cells were stained with recombinant CD22/Siglec-2–Fc, Siglec-7–Fc, and Siglec-9–Fc and analyzed with flow cytometry. Siglec–Fc binding signals (in MFI) were normalized to that of CD22/Siglec-2–Fc. Bars represent mean ± SD of three independent experiments. For primary B cells, the data was normalized individually for each donor [healthy donors (n = 9) and CLL patients (n = 17)], using the same dataset as presented in panel **(A)**. JVM-3 most closely resembled CLL B cells in terms of Siglec binding pattern. **(C)** Effect of sialidase treatment on Siglec-7 binding to JVM-3. JVM-3 cells were treated with (green) or without (red) sialidase before probing with recombinant Siglec-7–Fc. Siglec-7–Fc binding was abrogated by treatment of the cells with sialidase. Siglec-7(R124A)–Fc was used as a negative control (gray). **(D)** Effects of glycan processing inhibitors on Siglec-7–Fc binding to JVM-3. Cells were cultured in the presence of benzyl-2-acetamido-2-deoxy-α-d-galactopyranoside (benzyl-α-GalNAc; red: control; yellow: 0.2 mM; green: 0.5 mM; blue: 1 mM), kifunensine (red: control; yellow: 5 μM; green: 10 μM; blue: 20 μM), or dl-threo-1-phenyl-2-decanoylamino-3-morpholino-1-propanol (PDMP; red: control; yellow: 10 μM; green: 20 μM; blue: 40 μM) for 72 h; stained with recombinant Siglec-7–Fc; and analyzed with flow cytometry. Benzyl-α-GalNAc pretreatment attenuated Siglec-7–Fc binding, whereas neither kifunensine nor PDMP did, implying that O-glycans exhibit the glycan epitope (glycotope) recognized by Siglec-7. **(E)** Effect of O-sialoglycoprotein endopeptidase (OSGP-EP) treatment on Siglec-7–Fc binding to JVM-3. JVM-3 cells were treated with (green) or without (red) OSGP-EP before probing with recombinant Siglec-7–Fc. OSGP-EP treatment of JVM-3 cells attenuated Siglec-7–Fc binding, indicating that glycoproteins heavily modified by sialylated O-glycans (sialomucins) are the major ligands for Siglec-7. **(F)** Effect of enzyme treatment on Siglec-7–Fc binding to B cells from CLL patients (n = 5). Sialidase and OSGP-EP treatment of B cells from CLL patients diminished Siglec-7–Fc binding (****P < 0.0001, one-way ANOVA with Dunnett’s *post hoc* test). Bars represent mean ± SD.

**Figure 2:**
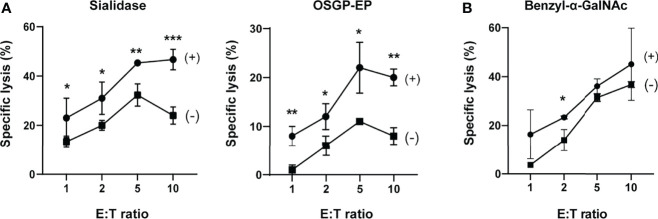
Sialylated O-glycoproteins protect chronic lymphocytic leukemia B cells from NK cell cytotoxicity. **(A)** Effects of sialidase or O-sialoglycoprotein endopeptidase (OSGP-EP) treatment of JVM-3 cells on NK cell cytotoxicity. JVM-3 cells were treated with sialidase or OSGP-EP and subjected to cytotoxicity assay using an NK-92 cell line expressing Siglec-7 (NK-92MI/S7). Both treatments made JVM-3 cells more sensitive to NK cell cytotoxicity. Cytotoxicity assays were conducted in technical triplicate and repeated several times, with consistent results. Representative results are shown (*P < 0.05, **P < 0.01, and ***P < 0.001; Student’s t test). Bars represent mean ± SD of technical triplicates. **(B)** Effect of benzyl-2-acetamido-2-deoxy-α-d-galactopyranoside (benzyl-α-GalNAc) treatment of JVM-3 on NK cell cytotoxicity. JVM-3 cells cultured in the presence of benzyl-α-GalNAc (72 h) were more sensitive to NK cell cytotoxicity. Cytotoxicity assays were conducted in technical triplicate and repeated several times, with consistent results. Representative results are shown (*P < 0.05, Student’s t test). Bars represent mean ± SD of technical triplicates.

**Figure 3:**
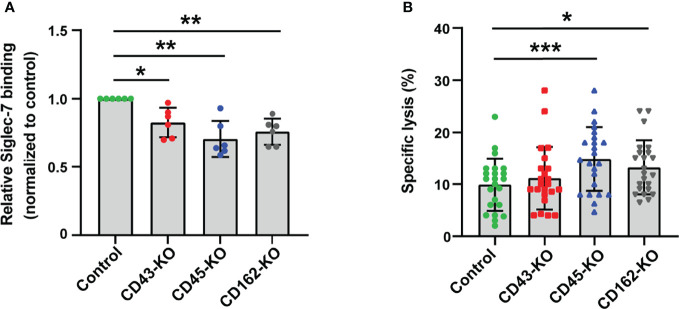
CD43, CD45, and CD162/PSGL-1 are the counterreceptors of Siglec-7. **(A)** Effect of glycoprotein knockout (KO) on Siglec-7–Fc binding. The glycoprotein genes (*SPN*, *PTPRC*, and *SELPLG* – encoding CD43, CD45, and CD162/PSGL-1, respectively) in JVM-3 were disrupted with CRISPR–Cas9 technology, and the cells were subjected to staining with Siglec-7–Fc. The disruption of individual genes led to a small but reproducible reduction in Siglec-7–Fc binding. Data was normalized by the Siglec-7–Fc binding (in MFI) to control JVM-3 cells. *P < 0.05, and **P < 0.01, one-way ANOVA with Dunnett’s *post hoc* test. Bars represent mean ± SD of 6 independent experiments. **(B)** Effect of glycoprotein KO on NK cell cytotoxicity. Glycoprotein KO and control JVM-3 cells were subjected to NK cell cytotoxicity assay. Disruption of CD45 and CD162/PSGL-1 led to increased sensitivity to NK cytotoxicity. A trend toward increased sensitivity of CD43 KO cells to NK cytotoxicity was observed, but it was not statistically significant (*P < 0.05 and ***P < 0.001, repeated-measures one-way ANOVA with Dunnett’s *post hoc* test). Bars represent mean ± SD of 23 independent experiments.

**Figure 4:**
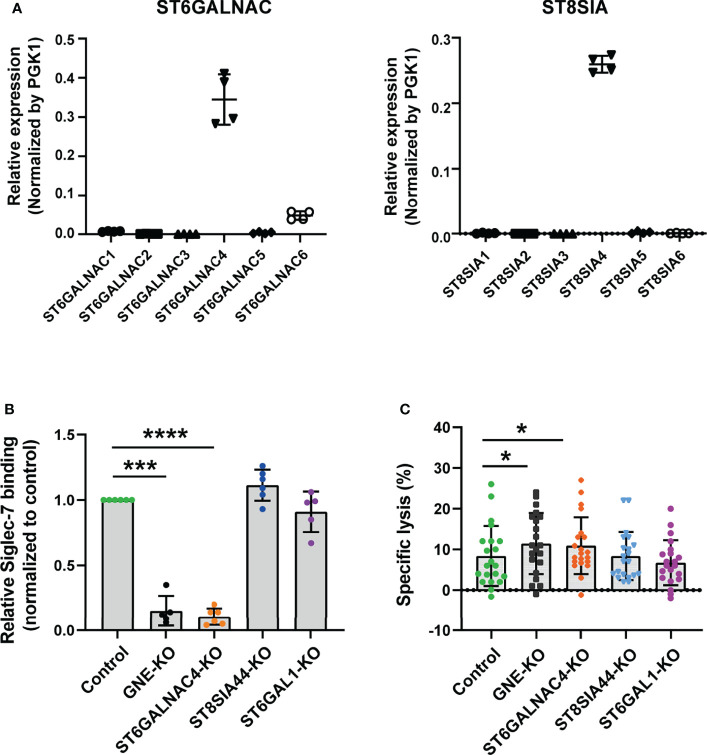
ST6GalNAc-IV is responsible for Siglec-7 ligand glycotope synthesis. **(A)** Sialyltransferases expressed in JVM-3. The transcript level for *ST6GALNAC4* was the highest among *ST6GALNAC*s, whereas that for *ST8SIA4* was the highest among *ST8SIA*s. Bars represent mean ± SD of technical quadruplicates. **(B)** Effect of sialyltransferase KO on Siglec-7–Fc binding. *GNE* and sialyltransferase genes (*ST6GALNAC4*, *ST8SIA4*, and *ST6GAL1*) in JVM-3 were disrupted with CRISPR–Cas9 technology, and the cells were subjected to staining with Siglec-7–Fc. The disruption of *GNE* and *ST6GALNAC4* led to a marked reduction in Siglec-7–Fc binding, whereas the disruption of *ST8SIA4* and *ST6GAL1* did not. Data was normalized by the Siglec-7–Fc binding (in MFI) to control JVM-3 cells. ***P < 0.001, and ****P < 0.0001, one-way ANOVA with Dunnett’s *post hoc* test. Bars represent mean ± SD of 6 independent experiments. **(C)** Effect of sialyltransferase KO on NK cell cytotoxicity. Sialyltransferase KO and control JVM-3 cells were subjected to NK cell cytotoxicity assay. The disruption of *GNE* and *ST6GALNAC4* led to increased sensitivity of JVM-3 cells to NK cytotoxicity, whereas the disruption of *ST8SIA4* and *ST6GAL1* did not (*P < 0.05, repeated-measures one-way ANOVA with Dunnett’s *post hoc* test). Bars represent mean ± SD of 21 independent experiments.

Statistical tests were performed with Prism 8 (GraphPad, San Diego, CA, USA) or with R. *P* value smaller than 0.05 was considered significant. Two-tailed tests were used throughout. For the comparison of two groups, Mann–Whitney test (when the normal distribution of values was not expected; **Figure 1A**) or Student’s t test (**Figures 2**, **4A**, **6C**) was used. For the comparison of the means of multiple groups, one-way ANOVA with Dunnett’s *post hoc* test (**Figures 1F**, **3C**, **4C**) was used. Association between gene expression and Siglec-7 binding (**Figure 6E**) was analyzed by linear regression, and that between gene expression and patient survival (**Figure 7** and **Table 1**) was analyzed by likelihood ratio test.

**Table 1:** Association of glycosyltransferase expression levels with the survival of patients with chronic lymphocytic leukemia.

Parameter	Hazard ratio (95% CI) by univariate analysis	Hazard ratio (95% CI) by multivariate analysis with age and *IGHV* mutation as covariates
Age (per year)	1.04 (1.01–1.07; *P* = 0.008)	1.04 (1.01–1.07; *P* = 0.015)
*IGHV* mutation (mutated/unmutated)	0.12 (0.06–0.24; *P* < 0.001)	0.15 (0.07–0.32; *P* < 0.001)
*GCNT1* (*G*) and *ST6GALNAC4* (*S*)		
*G* ^high^ *S* ^high^/*G* ^high^ *S* ^low^	3.25 (1.07–9.91; *P* = 0.038)	1.87 (0.59–5.96; *P* = 0.289)
*G* ^low^ *S* ^high^/*G* ^high^ *S* ^low^	7.61 (2.60–22.27; *P* < 0.001)	3.59 (1.16–11.12; *P* = 0.026)
*G* ^low^ *S* ^low^/*G* ^high^ *S* ^low^	4.62 (1.03–20.88; *P* = 0.045)	1.38 (0.27–7.00; *P* = 0.701)

Analyses of the association of GCNT1 and ST6GALNAC4 with mortality, with or without clinical covariates, were performed using International Cancer Genome Consortium data (n = 264) as described in Methods. The cutoff value for sample subgrouping was based on the optimal cutoff for gene expression (3.3 and 10.3 for GCNT1 and ST6GALNAC4, respectively) fitted in the Cox proportional hazards model. P values are based on likelihood ratio test. CI, confidence interval.

## Results

### B Cells From CLL Patients Express Higher Levels of Siglec-7 Ligands Than Those From Healthy Donors

Differences in the cellular or protein-specific glycosylation patterns between B cells from CLL patients and those from healthy donors have been described in the literature (38–42), but whether these changes alter interactions with Siglecs has not been specifically addressed to date. To compare the glycosylation profiles of B cells from CLL patients with those of B cells from healthy donors in the context of Siglec recognition, we tested the binding of several recombinant Siglecs to these cells by flow cytometry. We chose CD22/Siglec-2, Siglec-7, and Siglec-9 as probes, as these Siglecs showed robust binding to B cells from CLL patients in our preliminary experiments (data not shown). We found that B cells express ligands for several Siglecs and that B cells from CLL patients express higher levels of Siglec-7 ligands compared with those from healthy donors (**Figure 1A**). By contrast, the levels of ligands for CD22/Siglec-2 or Siglec-9 were not significantly different between the two groups (**Figure 1A**). The results for the CD22/Siglec-2 probe are consistent with those we obtained in a previous study, which demonstrated similar degrees of terminal α2–6 sialylation of N-glycans in B cells from CLL patients and healthy donors (42).

### Primary Siglec-7 Ligands in CLL B Cells Are O-Glycosylated Proteins

To investigate the molecular basis of Siglec-7 ligands in CLL B cells, we sought a CLL B cell line that resembles B cells from CLL patients in terms of glycan profile. Among the cell lines tested, JVM-3 showed a Siglec binding pattern similar to that of B cells from CLL patients (**Figure 1B**). Thus, we primarily used this cell line for further study.

As expected, sialidase treatment of JVM-3 cells diminished Siglec-7 binding (**Figure 1C**). Among the compounds that interfered with glycan processing, including benzyl-2-acetamido-2-deoxy-α-d-galactopyranoside (benzyl-α-GalNAc, mimicking the GalNAc peptide and diverting the O-glycan biosynthesis pathway), kifunensine (blocking N-glycan processing at high mannose–type glycans), and dl-threo-1-phenyl-2-decanoylamino-3-morpholino-1-propanol (inhibiting glycolipid biosynthesis), only benzyl-α-GalNAc significantly attenuated Siglec-7 binding to JVM-3 cells, suggesting that the glycotope on CLL B cells recognized by Siglec-7 is primarily exhibited on O-glycans (**Figure 1D**). We then treated the cells with O-sialoglycoprotein endopeptidase (OSGP-EP), which selectively digests mucin-like glycoproteins heavily modified with sialylated O-glycans (sialomucins) (43, 44). This treatment diminished Siglec-7 binding to JVM-3 cells (**Figure 1E**), demonstrating that glycoproteins heavily modified with O-glycans are the primary ligands for Siglec-7. Treatment of B cells from CLL patients with sialidase or OSGP-EP also diminished Siglec-7 binding (**Figure 1F**), confirming the observation with JVM-3.

### Enzymatic Removal of Sialylated O-Glycans Sensitizes JVM-3 to NK Cell Cytotoxicity

To test whether Siglec-7 ligands protect JVM-3 cells from NK cells, we enzymatically treated JVM-3 cells with sialidase or OSGP-EP and subjected them to NK cell cytotoxicity assay using the NK-92MI cell line expressing Siglec-7 (NK-92MI/S7). We over-expressed Siglec-7, as NK-92MI does not (or only weakly) express Siglec-7 (33).

As expected, both enzymatic treatments sensitized JVM-3 cells to NK cell cytotoxicity (**Figure 2A**). Moreover, the JVM-3 cell culture in the presence of benzyl-α-GalNAc also sensitized the cells to NK cell cytotoxicity (**Figure 2B**). Taken together, these results imply that sialylated glycotopes on heavily O-glycosylated proteins (counterreceptors) protect CLL B cells from NK cell cytotoxicity. We observed a similar enhancement of cytotoxicity by the same treatment of JVM-3 cells when parental NK-92MI cells were used as effector cells (**Supplementary Figure 2**), implying that sialylated and heavily O-glycosylated proteins can protect CLL by a Siglec-7-independent mechanism as well. Although we found Siglec-6 is highly expressed on parental NK-92MI, recombinant Siglec-6 did not show binding to JVM-3 (**Supplementary Figure 3**), excluding the interaction between NK-92MI and JVM-3 cells by way of Siglec-6 and its ligand.

### Siglec-7 Counterreceptors on CLL B Cells Include CD43, CD45, and PSGL-1

We used a proximity biotin labeling method (32) to identify the counterreceptors for Siglec-7 and determined CD45 as a candidate (**Supplementary Dataset 1**). CD43, a major sialomucin, was also identified with a single peptide. However, other sialomucins (e.g., CD162/P-selectin glycoprotein ligand-1 [PSGL-1]) were not identified, likely because these proteins are resistant to proteolysis and inherently difficult to identify by mass spectrometry (45).

Flow cytometry analysis revealed that CD43 and CD162/PSGL-1 are expressed on JVM-3 cells (data not shown). We thus tested whether either of these proteins or CD45 accounts for a major counterreceptor by knocking out each of them. Gene disruption (*SPN* for CD43, *PTPRC* for CD45, and *SELPLG* for CD162/PSGL-1) revealed that none of these proteins alone could account for the Siglec-7 counterreceptor but that depletion of each glycoprotein attenuated the Siglec-7 binding to a small extent (**Figure 3A**).

We then tested whether any of the knockout cells show increased sensitivity to NK cell cytotoxicity. As expected, cells deficient in CD45 or CD162/PSGL-1 were more sensitive to cytolysis by NK-92MI/S7 (**Figure 3B**). Taken together, these results indicate that CD45 and CD162/PSGL-1 are functional Siglec-7 counterreceptors on CLL B cells.

### Siglec-7 Glycotope on CLL B Cells Is Synthesized by ST6GalNAc-IV

To gain further insight into the glycan part of Siglec-7 ligands, we sought the sialyltransferase responsible for the biosynthesis of the glycotope recognized by Siglec-7. Siglec-7 preferentially recognizes α2–8–linked oligosialic acids ([Neu5Acα2–8]n; n ≧ 2), disialyl N-acetyllactosamine (Neu5Acα2–3Galβ1–4[Neu5Acα2–6]GlcNAcβ1–), and a terminal tetrasaccharide of α-series gangliosides (Neu5Acα2–3Galβ1–3[Neu5Acα2–6]GalNAcβ1–) (46–51), which are elaborated by ST8Sia and the ST6GalNAc family of sialyltransferases, respectively. Therefore, we analyzed the expression profiles of these sialyltransferases in the JVM-3 cell line; we found that *ST8SIA4* and *ST6GALNAC4* were highly expressed (**Figure 4A**). As shown in **Figure 4B**, *ST6GALNAC4*-deficient cells showed a clear reduction in Siglec-7 binding, whereas those deficient in *ST8SIA4* or *ST6GAL1* did not. As expected, JVM-3 cells deficient in *GNE* (encoding UDP-GlcNAc 2-epimerase/ManNAc 6-kinase, the first enzyme in the sialic acid biosynthesis pathway) also showed a clear reduction in Siglec-7 binding (**Figure 4B**).

To test whether the JVM-3 cells deficient in Siglec-7 glycotope are more sensitive to NK cell cytotoxicity, we subjected the cells to cytotoxicity assay. As expected, *ST6GALNAC4* and *GNE* deficient cells were more sensitive to NK cell cytotoxicity than the control cells were, whereas *ST8SIA4* and *ST6GAL1* deficient cells were not (**Figure 4C**). Taken together, these results indicate that *ST6GALNAC4* is responsible for the biosynthesis of the glycotope that protects CLL B cells from NK cell cytotoxicity.

### The disialyl-T Structure Is the CLL Glycotope Recognized by Siglec-7

**Figure 5:**
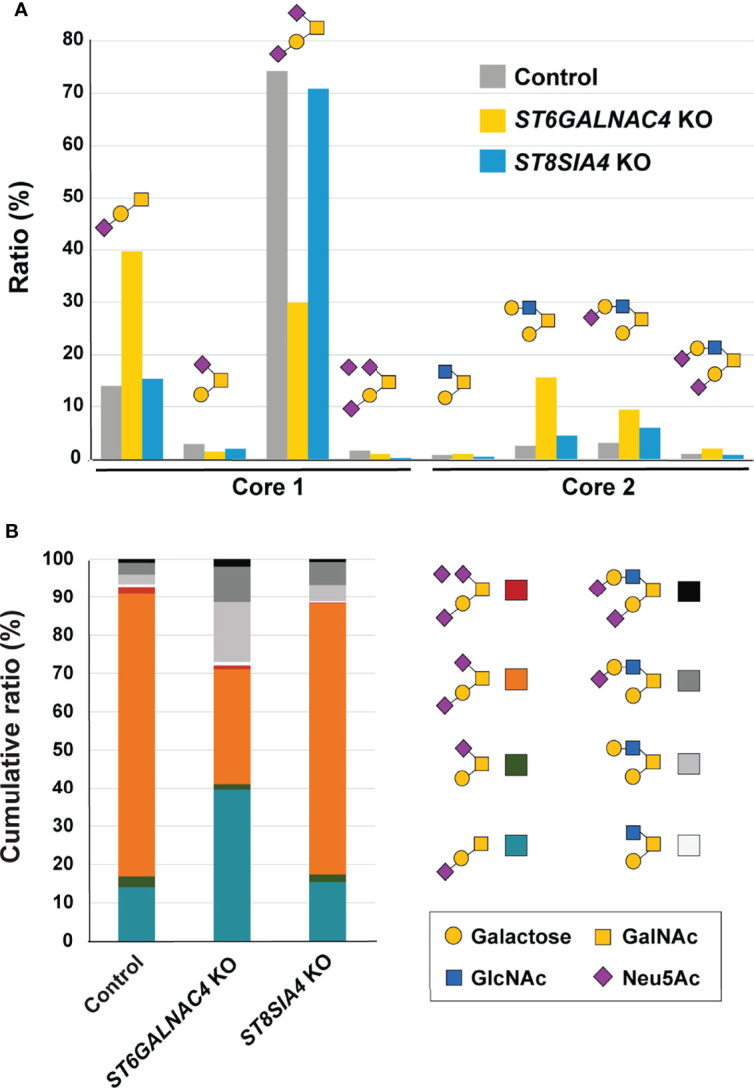
ST6GalNAc-IV is responsible for the biosynthesis of disialyl-T in JVM-3 cells. **(A)** O-glycans were released by reductive elimination from control (gray), *ST6GALNAC4* KO (yellow), and *ST8SIA4* KO (blue) JVM-3 cells; permethylated; and subjected to liquid chromatography with tandem mass spectrometry analysis. Except for monosialyl-T (Neu5Acα2–3Galβ1–3GalNAcα1– or Galβ1–3[Neu5Acα2–6]GalNAcα1–), which could be resolved by liquid chromatography into two distinct isomeric structures, and trisialyl-T, which consisted of two unresolved positional isomers (Neu5Acα2–3Galβ1–3[Neu5Acα2–8Neu5Acα2–6]GalNAcα1– and Neu5Acα2–8Neu5Acα2–3Galβ1–3[Neu5Acα2–6]GalNAcα1–), each of the other O-glycans was found to be represented by a single dominating structure, as determined by tandem mass spectrometry and annotated accordingly using the Symbol Nomenclature for Glycans (52). Relative abundance was calculated from the peak areas of extracted ion chromatograms and normalized to the percentage total. Disruption of *ST6GALNAC4* resulted in a reduction in the disialyl-T (Neu5Acα2–3Galβ1–3[Neu5Acα2–6]GalNAcα1–) structure and a concomitant increase in the monosialyl-T (Neu5Acα2–3Galβ1–3GalNAcα1–) and core 2 (e.g., Galβ1–3[Galβ1–3GlcNAcβ1–6]GalNAcα1–) structures. Disruption of *ST8SIA4* resulted in the loss of the trisialyl-T structure. **(B)** Stacked bar chart of the same data shown in panel **(A)**, along with the color code used for each of the eight major O-glycans identified and quantified.

To determine the glycotope elaborated by ST6GalNAc-IV, we subjected the control and *ST6GALNAC4*-deficient JVM-3 cells to quantitative O-glycan analysis by liquid chromatography–tandem mass spectrometry (LC–MS/MS). As shown in **Figure 5**, control JVM-3 cells predominantly expressed variably sialylated core 1 O-glycan (Galβ1–3GalNAcα1–) structures, with disialyl-T (Neu5Acα2–3Galβ1–3[Neu5Acα2–6]GalNAcα1–) being the most abundant. By contrast, *ST6GALNAC4*-deficient JVM-3 cells showed a significant loss of disialyl-T as well as a concomitant increase in monosialyl-T (Neu5Acα2–3Galβ1–3GalNAcα1–) and core 2 O-glycan structures (e.g., Galβ1–3[Galβ1–4GlcNAcβ1–6]GalNAcα1–). These results strongly suggest that disialyl-T is the primary glycotope on CLL B cells recognized by Siglec-7. We noticed that the trisialyl-T structure (Neu5Acα2–3Galβ1–3[Neu5Acα2–8Neu5Acα2–6]GalNAcα1– and/or Neu5Acα2–8Neu5Acα2–3Galβ1–3[Neu5Acα2–6]GalNAcα1–) was reduced in *ST6GALNAC4*-deficient JVM-3 cells and further diminished in *ST8SIA4*-deficient cells, suggesting that disialyl-T serves as an acceptor substrate for ST8Sia-IV. Nevertheless, as *ST8SIA4* deficiency neither impaired Siglec-7 binding nor enhanced NK cell cytotoxicity, O-glycans with linear oligosialic acids do not appear to be essential for Siglec-7 binding or the resistance of CLL B cells to NK cell cytotoxicity.

### Expression of GCNT1 Interferes With the Biosynthesis of Siglec-7 Ligands

**Figure 6:**
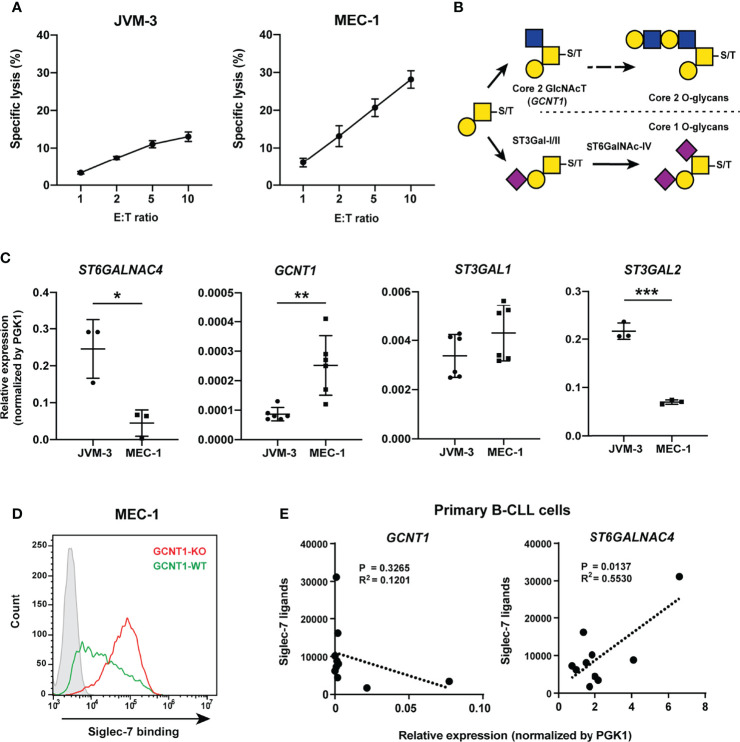
Core 2 GlcNAc transferase interferes with the biosynthesis of the glycotope recognized by Siglec-7. **(A)** NK cell cytotoxicity assay of JVM-3 and MEC-1 cell lines. JVM-3 cells were more resistant than MEC-1 cells to NK cell cytotoxicity. Cytotoxicity assays were conducted in technical triplicate and repeated several times, with consistent results. Representative results are shown. Bars represent mean ± SD of technical triplicates. **(B)** Schematic representation of O-glycan biosynthesis in leukocytes. **(C)** Quantitative real-time polymerase chain reaction analysis of glycosyltransferases in JVM-3 and MEC-1 cells. *ST6GALNAC4* expression was higher in JVM-3, whereas *GCNT1* expression was higher in MEC-1 (*P < 0.05, **P < 0.01, and ***P < 0.001; Student’s t test). Bars represent mean ± SD of technical replicates (n = 3–6). **(D)** Effect of *GCNT1* disruption in MEC-1 cells on Siglec-7–Fc binding. **(E)** Correlation of the *GCNT1* and *ST6GALNAC4* transcript levels and Siglec-7–Fc binding (in median fluorescence intensity [MFI]) to B cells from patients with chronic lymphocytic leukemia (n = 10). Association between gene expression and Siglec-7 binding was analyzed by linear regression.

MEC-1 cells are more sensitive to NK cell cytotoxicity compared with JVM-3 cells (**Figure 6A**), which coincided with weaker Siglec-7 binding (**Figure 2A**). Given that (i) the addition of GlcNAc at C6 of GalNAc by core 2 GlcNAc transferase (encoded by *GCNT1*) precludes the sialylation at the same position by ST6GalNAc-IV (**Figure 6B**) and (ii) the expression level of *GCNT1* in MEC-1 is higher than that in JVM-3 (**Figure 6C**), we speculated that the expression of *GCNT1* interferes with the expression of Siglec-7 glycotope in MEC-1 cells. As expected, *GCNT1* disruption in MEC-1 cells enhanced Siglec-7 binding (**Figure 6D**). To confirm the effects of *GCNT1* and *ST6GALNAC4* on Siglec-7 ligand expression, we quantified their transcript levels by qRT-PCR and analyzed their association with Siglec-7 ligand levels on B cells from CLL patients. As expected, high expression of *GCNT1* was associated with weaker Siglec-7 binding, whereas the expression of *ST6GALNAC4* showed a positive correlation with Siglec-7 binding (**Figure 6E**).

### High Expression of ST6GALNAC4 and Low Expression of GCNT1 Are Associated With Poor Prognosis in CLL Patients

**Figure 7:**
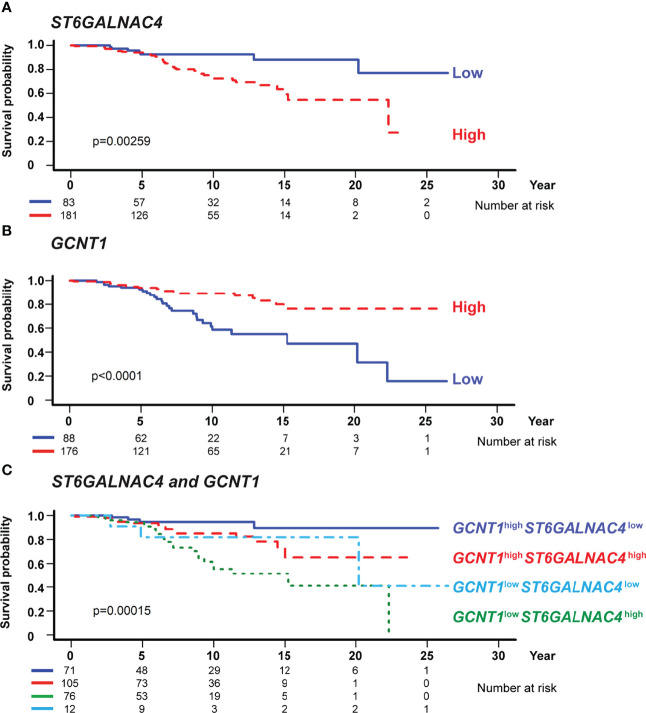
GCNT1 and ST6GALNAC4 expression levels are associated with the prognosis of patients with chronic lymphocytic leukemia (CLL). Shown are the Kaplan–Meier survival plots with logrank test for two subgroups dichotomized with the expression levels of *ST6GALNAC4* **(A)** and *GCNT1* **(B)** as well as for four subgroups with the expression levels of both genes **(C)** using the optimal cutoff fitted in the Cox proportional hazards model. High expression of *ST6GALNAC4* **(A)** and low expression of *GCNT1* **(B)** were associated with poorer prognosis in CLL patients (*P* = 0.00259 and < 0.0001, respectively; likelihood ratio test). In panel **(C)**, the survival curves of four subgroups are significantly different (*P* = 0.00015), and the prognosis of *GCNT1* ^low^ *ST6GALNAC4* ^high^ patients was significantly poorer as compared with that of *GCNT1* ^high^ *ST6GALNAC4* ^low^ patients (*P* < 0.001, likelihood ratio test; see also **Table 1**).

To test whether the expression levels of *ST6GALNAC4* and *GCNT1* show any association with the prognosis of CLL patients, we analyzed the correlations between the overall survival of CLL patients and the expression levels of these genes using the CLL RNA sequencing data set in the International Cancer Genome Consortium database (37, 53). RNA sequencing data of the patients with CLL or small cell lymphoma and with survival data (n = 255 and n = 9, respectively; total n = 264) were included in the analysis. Our analysis revealed that high *ST6GALNAC4* expression and low *GCNT1* expression are associated with poor prognosis (**Figure 7A, B**, respectively). Moreover, by comparing four groups of patients stratified by *ST6GALNAC4* and *GCNT1* expression levels, we found that the prognosis of *GCNT1* ^low^ *ST6GALNAC4* ^high^ patients is the least favorable (overall, *P* = 0.00015; *GCNT1* ^low^ *ST6GALNAC4* ^high^ vs. *GCNT1* ^high^ *ST6GALNAC4* ^low^ groups, *P* < 0.001; **Figure 7C**). This association remained significant even when age and *IGHV* mutation status (a strong prognostic factor for CLL) were included as covariates (*P* = 0.026; **Table 1**). Taken together, these results suggest that the expression of the disialyl-T structure is associated with poor prognosis in CLL patients, possibly through immunoevasion by engagement of Siglec-7 on NK cells.

## Discussion

In this study, we demonstrated that B cells from CLL patients express higher levels of Siglec-7 ligands compared with those from healthy donors and that the ligands protect B cells from NK cell cytotoxicity. The glycotope recognized by Siglec-7 is the disialyl-T (Neu5Acα2–3Galβ1–3[Neu5Acα2–6]GalNAcα1–) structure, which was exhibited on various counterreceptors, including CD43, CD45, and CD162/PSGL-1. The glycan epitope was synthesized by ST6GalNAc-IV (encoded by *ST6GALNAC4*), and its synthesis was blocked by core 2 GlcNAc transferase (encoded by *GCNT1*). The expression levels of these two glycosyltransferases were associated with the overall survival of CLL patients, and the pattern predictive of high disialyl-T expression (*GCNT1* ^low^ *ST6GALNAC4* ^high^) was associated with poor prognosis. These data imply that the mechanism underlying the poor prognosis in *GCNT1* ^low^ *ST6GALNAC4* ^high^ patients likely involves the high expression of the disialyl-T structure, which may facilitate immunoevasion by engaging Siglec-7 on NK cells.

The O-glycosylation pattern of human B cells has been previously reported to change during differentiation, and a reduction in *GCNT1* expression and a concomitant shortening of O-glycans were observed in the cells that have undergone germinal center reaction (54). Research has also shown that the level of Siglec-7 ligands on human B cells changes during differentiation, with naive and memory cells expressing high levels of Siglec-7 ligands, whereas it decreases temporarily on activated naive cells (55). Therefore, the expression level of Siglec-7 ligands potentially reflects the differentiation stage of the B-cell clone that gave rise to CLL. However, our analysis (data not shown) indicated that *GCNT1* expression is higher in *IGHV*-mutated CLL (reflecting somatic hypermutation in germinal center), which is opposite of our expectation [i.e., *GCNT1* expression diminishes during B-cell maturation (54)]. Regardless, when the *IGHV* mutation status was included in the multivariate analysis, the association between overall survival and the *ST6GALNAC4* and *GCNT1* expression levels remained significant (**Table 1**). Thus, *ST6GALNAC4* and *GCNT1* transcription and disialyl-T expression levels may serve as independent criteria for the prognosis of CLL patients.

The observed association of *GCNT1* expression with CLL prognosis is incongruent with data reported for solid tumors (e.g., bladder and prostate cancers), in which high expression of *GCNT1* was associated with poor prognosis, presumably through the protection of tumors from NK cells (56–58). We speculate that this discrepancy can be attributed to the difference in the lectins and counterreceptors involved. The extension of polylactosamine on the core 2 O-glycans on major histocompatibility complex class I polypeptide–related sequence A (MICA) was found to reduce its binding with the cognate receptor NK group 2 member D (NKG2D) on NK cells, both directly and by way of binding with galectin-3 (58). The cell lines we used (JVM-3 and MEC-1) expressed low levels of MICA (data not shown). MICA was slightly upregulated on B cells from CLL patients compared with those from healthy donors, whereas high plasma levels of soluble NKG2D ligands (soluble MICA, MICB, and UL16 binding protein 2) were associated with poor treatment-free survival of CLL patients (59), suggesting that soluble NKG2D ligands may compromise NKG2D-mediated NK cell activation in CLL. In addition, as the polylactosamine extension on O-glycans on B cells is limited (data not shown), interruption of the MICA–NKG2D interaction by polylactosamine may not play a major role in CLL. Regardless, the difference in the role of *GCNT1* between solid tumors and CLL underscores the importance of understanding the nature of the glycotope and counterreceptors serving as ligands for Siglecs.

Two recent studies independently identified CD43 as a Siglec-7 counterreceptor on the K562 erythroleukemia cell line, which is often used as a target for NK cytotoxicity assays, and demonstrated that knockout/knockdown of CD43 renders K562 cells more sensitive to NK cytotoxicity (34, 60). By contrast, our analysis revealed that CD43 is not the sole counterreceptor for Siglec-7. The difference between K562 and CLL may be explained by the different repertoires of glycoproteins expressed on these cells. For instance, K562 cells express CD43, but not CD162/PSGL-1, at high levels (data not shown). Another recent study, investigating the resistance of multiple myeloma cells to NK cytotoxicity, revealed that multiple myeloma cells express high level of Siglec-7 ligand, and CD162/PSGL-1 is a major Siglec-7 counterreceptor on multiple myeloma cells (61). Yet another recent study using a genetically manipulated HEK293T cell line demonstrated that *GCNT1* and *ST6GALNAC*s regulate the expression of Siglec-7 glycotope, which the authors deduced to be disialyl-T, and found a strong dependence of Siglec-7 binding on the type of counterreceptor expressed (62).

NK cells in CLL patients have been reported to be functionally impaired (63). Although the subject is beyond the scope of this study, Siglec-7 ligands on CLL B cells can be hypothesized to induce the state of NK cell exhaustion by engaging Siglec-7. If this is true, then blocking the interaction between Siglec-7 on NK cells and its ligands on CLL B cells may restore the cytotoxic activity of NK cells.

Our study has some limitations. Although our analysis of Taiwanese CLL patient samples found correlations between *GCNT1* and *ST6GALNAC4* expression levels and Siglec-7 glycotope (**Figure 6E**), the findings are not definitive because of the limited number of samples. Moreover, the clinical benefit of glycotope testing remains unknown. A prospective study enrolling more patients would address this issue better.

## Data Availability Statement

The RNA-sequencing datasets presented in this article are not readily available, because the dataset is International Cancer Genome Consortium (ICGC) Controlled Data, and the Data Access Agreement between ICGC and the PI does not permit the transfer or disclosure of any material derived from the ICGC Controlled Data to anyone not listed in the data access application. Requests to access the datasets should be directed to the Data Access Control Office of ICGC (https://daco.icgc-argo.org). The proteomic data is publicly available in the PRIDE database (accession no. PXD024690).

## Ethics Statement

The studies involving human participants were reviewed and approved by National Taiwan University Hospital and Academia Sinica. The patients/participants provided their written informed consent to participate in this study.

## Author Contributions

L-YC, HCT, S-CL, H-YT, C-JT, MS, and Yi-JC performed the experiments and analyzed the data. S-YL analyzed the CLL transcriptomic data. S-JW recruited patients and collected patient blood samples. Yu-JC, K-IL, K-HK, and TA designed the research, analyzed the data, and wrote the manuscript. All authors contributed to the article and approved the submitted version.

## Funding

This study was financially supported by Academia Sinica Thematic Project grants (AS-105-TP-A05 and AS-TP-108-ML06). The National Core Facility for Biopharmaceuticals (see acknowledgment) is supported by the Ministry of Science and Technology (MOST 106-2319-B-492-002). The Academia Sinica Common Mass Spectrometry Facilities for Proteomics and Protein Modification Analysis (see acknowledgment) is supported by an Academia Sinica Core Facility and Innovative Instrument Project grant (AS-CFII-108-107).

## Conflict of Interest

The authors declare that the research was conducted in the absence of any commercial or financial relationships that could be construed as a potential conflict of interest.

## Publisher’s Note

All claims expressed in this article are solely those of the authors and do not necessarily represent those of their affiliated organizations, or those of the publisher, the editors and the reviewers. Any product that may be evaluated in this article, or claim that may be made by its manufacturer, is not guaranteed or endorsed by the publisher.
