# Supplementary material for: Molecular Basis and Role of Siglec-7 Ligand Expression on Chronic Lymphocytic Leukemia B Cells
Source: Front Immunol. 2022 May 31;13:840388. doi: 10.3389/fimmu.2022.840388 (PMC9195294; doi:10.3389/fimmu.2022.840388)
Supplement: Supplementary file 1 [file DataSheet_1.pdf]

Supplementary Material

Supplementary Materials and Methods

Reagents

Biotinylated *Maackia amurensis* lectin-II (MAL-II) was obtained from Vector Laboratories (Burlingame, CA, USA). Phycoerythrin-labeled streptavidin was purchased from Jackson ImmunoResearch (West Grove, PA, USA). Anti-oligoNeu5Ac monoclonal antibody (clone 2-2B) was from Merck Millipore (Darmstadt, Germany).

Proximity labeling of JVM-3 cells with Siglec-7-Fc and identification of counter receptor candidates with mass spectrometry-based proteomics

Identification of Siglec-7 counterreceptors was attempted with proximity labeling as described previously (1). In brief, JVM-3 cells (1×10^7 cells) were incubated with Siglec-7-Fc (10 µg) or binding-deficient mutant Siglec-7(R124A)-Fc (10 µg) precomplexed with peroxidase-conjugated anti-FLAG M2 antibody (5 µg; cat. no. A8592, Sigma) on ice for 30 min. The cells were washed with Tris-buffered saline (TBS; 20 mM Tris-HCl, pH 7.5, 140 mM NaCl) and further incubated with labeling reagent (10 µM biotin tyramide, 10 mM H_2O_2 in TBS) at room temperature for 10 min. The cells were washed with TBS, lysed with lysis buffer (50 mM Tris-HCl buffer, pH 8.0, 150 mM NaCl, 1% Nonidet P-40, 0.5% sodium deoxicholate, 0.1% sodium dodecyl sulfate (SDS), and protease inhibitor cocktail), and biotinylated proteins were purified with Dynabeads MyOne Streptavidin C1 beads (Thermo Fisher Scientific). Captured proteins were eluted by heat denaturation in sample buffer and subjected to short SDS-polyarcylamide gel electrophoresis (SDS-PAGE). Gel areas containing proteins were excised and subjected to in-gel trypsin digestion, as described previously (1). The peptides were extracted, desalted by C18 Zip-tip (Millipore), dissolved in 0.1% formic acid in water and subjected to a nanoAcquity system (Waters, Milford, MA) connected to the Orbitrap Elite hybrid mass spectrometer (Thermo Electron, Bremen, Germany) equipped with a PicoView nanospray interface (New Objective, Woburn, MA). Liquid chromatography was performed with a C18 BEH column with 75 µm ID, 25 cm length (Waters, Milford, MA, USA) packed with 1.7 µm particles with a pore size of 130 Å, with a segmented gradient in 120 min from 5% to 35% solvent B (acetonitrile with 0.1% formic acid) at a flow rate of 300 nl/min and a column temperature of 35 °C. The mass spectrometer was operated in the data-dependent mode. Briefly, precursor ion scanning was set to 60K at m/z 400 and automatic gain control (AGC) target at 10^6. The 20 most intense ions with charge states ≥2 were sequentially isolated for CID MS/MS fragmentation with normalized collision energy of 35% and detection in the linear ion trap (AGC target at 10000) with previously selected ions dynamically excluded for 60 s. Ions with singly and unrecognized charge state were also excluded.

The raw data were processed using Proteome Discoverer 2.1 (Thermo Fisher Scientific), and peptide identification was performed by Mascot (version 2.3.2) and SEQUEST search engines against the Swiss-Prot database (v2015_12, total 20,193 sequences from human) with a percolator (strict false discovery rate (FDR) of 0.01 and a relaxed FDR of 0.05). The parameter of protein and peptide identification was set as: trypsin with 2 maximum missing cleavage sites, 10 ppm for mass tolerance for precursor ion mass, 0.1 Da for the fragment ion tolerance, and variable modifications including oxidation at methionine (M), carbamidomethyl at cysteine (C), and deamidation at asparagine (N) or glutamine (Q). Label-free quantification was performed using the peak area of each precursor ion calculated from extracted ion chromatogram (XIC) during data processing using the Precursor Ions Area Detector node with mass precision 2 ppm. The abundance of identified protein was calculated from the top three of all unique and razor peptides in Peptide and Protein Quantifier node, and was used to calculate the relative protein abundance between experimental samples. Proteins that were 10 times or more abundant in the labeled sample compared with control sample were considered Siglec-7 ligand candidates. Proteomics dataset was deposited to ProteomeXchange via the PRIDE database (accession number: PXD024690).

Cellular O-glycome analysis by LC-MS/MS

Proteins extracted from 1×10^7 harvested JVM-3 cells by sonication in 1% of Triton X-100 were subjected to reduction and alkylation by 10 mM of dithiothreitol and 50 mM of iodoacetic acid for 1 h at 37 °C, and then precipitated in trichloroacetic acid at a final concentration of 10%. The protein pellets were washed by cold acetone to remove detergents and then reconstituted in 50 mM ammonium bicarbonate buffer for overnight trypsin digestion at 37 °C, followed by chymotrypsin at 37 °C for 8 h, and overnight PNGase F treatment at 37 °C twice to release the N-glycans. O-glycans were subsequently released by reductive elimination (1 M of $NaBH_4$ in 50 mM of NaOH) at 45 °C for 16 h, after which the reaction mixtures were neutralized, loaded onto a C18 Sep-Pak cartridge (Waters) pre-packed with 1 ml of activated Dowex 50W-X8 resin, and the reduced glycans eluted by 5% AcOH. After removal of borates by repeated co-evaporation with 10% acetic acid in methanol, the reduced glycan samples were permethylated and subjected to LC–MS/MS analysis on an Orbitrap Fusion™ Tribrid™ Mass Spectrometer (Thermo Fisher Scientific), as described previously (2), but using instead a ReproSil-Pur 120 C18-AQ column (120Å, 1.9 µm, 75 µm × 200 mm; Dr. Maisch HPLC GmbH, Ammerbuch, Germany) without altering any of the LC conditions and data acquisition methods.

Data analysis were performed manually using the Xcalibur software v2.3. The ion chromatograms of commonly found cores 1 and 2 O-glycans were extracted at 10 ppm for either singly or doubly protonated molecular ions depending on their molecular masses and the exact species detected. The structures of resolved peaks were then assigned by manual interpretation of their corresponding MS/MS data. Each of the identified O-glycan structures was relatively quantified based on the verified peak areas of their respective extracted ion chromatograms and expressed as % total of all quantified O-glycan peaks derived from that sample.

International Cancer Genome Consortium (ICGC) CLL transcriptomic data analysis

RNAseq-based transcriptomic datasets for CLL patients (EGAD00001000258 and EGAD00001001443) were downloaded from ICGC data repository, and analyzed using Taiwania 1 supercomputer at National High-performance Computing Center (Hsinchu, Taiwan) and GNU parallel computing (3). RNAseq data for the patients with CLL or small lymphocytic lymphoma and with survival status (n = 255 and n = 9, respectively; total: n = 264) were included in the subsequent analysis, while those for healthy donors, donors with monoclonal B lymphocytosis, and CLL patients without survival status were excluded. Sequencing quality was checked first with fastQC v0.11.8 (4), and paired-end sequencing reads were mapped to human genome (GRCh38) and quantified with Salmon v. 1.0 using quasi-mapping mode, fragment GC bias and positional bias correction, and other default settings (5). The transcriptome count matrices from Salmon were further processed with tximport (6) and DESeq2 (7) to the gene level with an average transcript length offset which was used to control the bias of transcript length using the GLM approach (8). Gene counts greater than 5 and the frequency in the samples greater than 5% were retained for statistical analysis. The gene counts were further normalized with the sample-specific size factors determined by median ratio of gene counts relative to geometric mean per gene across samples using the counts function from DESeq2 to control the bias in sample variation.

The normalized gene expression matrix from aforementioned results was log2 transformed and analyzed for differential gene expression using DESeq2 with respect to *IGHV* mutation status (9). The data also was analyzed using Chi-square test, Linear logistic regression with binomial distribution, and Cox proportional hazards model using finalfit (10), a R package, with or without clinical variables to access the association of gene expression and/or clinical variable with mortality. The cutoffs for dichotomizing sample group by gene expression for the association analysis included mean, median, and the optimal cutoff in the Cox proportional hazards model using the cutp function in survMisc (11), a R package. Optimal cutoff determines the value to spilt the group so that it yields the lowest p value with log-rank test for the two groups fitted in the survival model (12).

References

1. Chang L, Chen YJ, Fan CY, Tang CJ, Chen YH, Low PY, et al. Identification of Siglec Ligands Using a Proximity Labeling Method. J Proteome Res. 2017;16(10):3929-41.

2. Hsiao CT, Wang PW, Chang HC, Chen YY, Wang SH, Chern Y, et al. Advancing a High Throughput Glycotope-centric Glycomics Workflow Based on nanoLC-MS(2)-product Dependent-MS(3) Analysis of Permethylated Glycans. Mol Cell Proteomics. 2017;16(12):2268-80.

3. Tange O. GNU Parallel 2018. 2018.

4. Andrews S. FastQC. Ver. 0.11.8. ed2018.

5. Patro R, Duggal G, Love MI, Irizarry RA, Kingsford C. Salmon provides fast and bias-aware quantification of transcript expression. Nat Methods. 2017;14(4):417-9.

6. Soneson C, Love MI, Robinson MD. Differential analyses for RNA-seq: transcript-level estimates improve gene-level inferences. F1000Res. 2015;4:1521.

7. Anders S, Huber W. Differential expression analysis for sequence count data. Genome Biol. 2010;11(10):R106.

8. Love MI, Soneson C, Patro R. Swimming downstream: statistical analysis of differential transcript usage following Salmon quantification. F1000Res. 2018;7:952.

9. Puente XS, Bea S, Valdes-Mas R, Villamor N, Gutierrez-Abril J, Martin-Subero JI, et al. Non-coding recurrent mutations in chronic lymphocytic leukaemia. Nature. 2015;526(7574):519-24.

10. Harrison E, Drake T, Ots R. finalfit: Quickly create elegant regression results tables and plots when modelling. Ver. 1.0.2. ed2020.

11. Dardis C. survMisc: Miscellaneous functions for survival data. Ver. 0.5.5. ed2018.

12. Contal C, O'Quigley J. An application of changepoint methods in studying the effect of age on survival in breast cancer. Computational Statistics & Data Analysis. 1999;30(3):253-70.

A

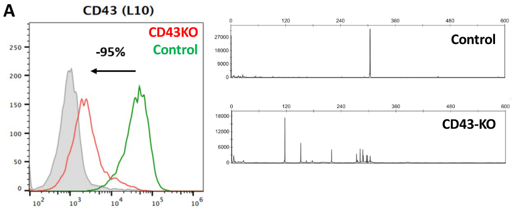

Sample	Size (nt)	Indel (nt)	Area	%
Control	304.7		285491	100
CD43-KO	117.0	-188	90970	27
	151.7	-153	41318	12
	219.2	-85	29433	9
	273.8	-31	20878	6
	275.8	-29	11433	3
	281.7	-23	32815	10
	287.7	-17	32942	10
	288.7	-16	19599	6
	295.7	-9	16857	5
	297.8	-7	16192	5
	303.8	-1	21040	6

B

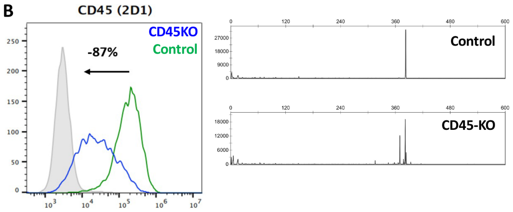

Sample	Size (nt)	Indel (nt)	Area	%
Control	382.2	0	355651	100
CD45-KO	315.9	-66	11242	4
	369.8	-12	93836	30
	377.8	-4	13505	4
	381.6	-1	156460	50
	383.6	1	35166	11

C

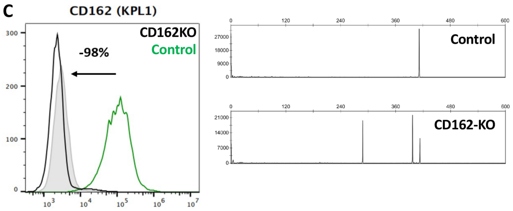

Sample	Size (nt)	Indel (nt)	Area	%
Control	411.7	0	398037	100
CD162-KO	288.0	-124	126018	32
	396.9	-15	178510	45
	413.0	1	92900	23

D

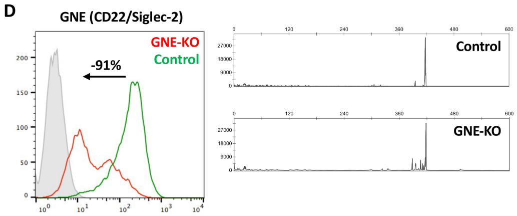

Sample	Size (nt)	Indel (nt)	Area	%
Control	395.4	-22	33976	6
	417.2	0	503067	85
	418.5	1	53321	9
GNE-KO	388.3	-29	88883	11
	395.5	-22	52431	6
	396.3	-21	34636	4
	406.5	-11	69681	9
	410.4	-7	42644	5
	413.3	-4	27335	3
	416.4	-1	90892	11
	418.2	1	413203	50

E

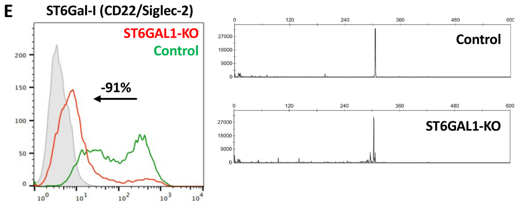

Sample	Size (nt)	Indel (nt)	Area	%
Control	305.3	0	323251	100
ST6G1-KO	296.2	-9	63368	11
	297.1	-8	26548	5
	303.5	-2	393100	71
	304.9	0	16695	3
	306.1	1	17379	3
	307.0	2	38588	7

F

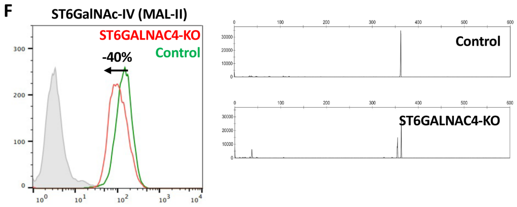

Sample	Size (nt)	Indel (nt)	Area	%
Control	361.9	0	393918	100
ST6GN4-KO	325.6	-36	9533	2
	342.7	-19	5770	1
	354.1	-8	82889	14
	355.0	-7	153876	28
	363.2	1	303547	55

G

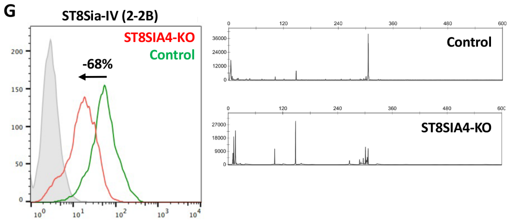

Sample	Size (nt)	Indel (nt)	Area	%
Control	301.1	-4	26571	6
	305.6	0	426090	94
ST8SIA4-KO	266.1	-39	40310	10
	288.3	-17	29396	7
	296.3	-9	38888	10
	301.2	-4	99197	25
	303.1	-2	27791	7
	304.1	-1	24858	6
	305.2	0	47372	12
	307.1	2	88979	22

H

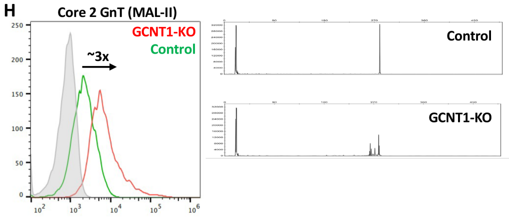

Sample	Size (nt)	Indel (nt)	Area	%
Control	279.6	0	275822	100
GCNT1-KO	262.5	-17	15911	6
	263.5	-16	45418	17
	265.4	-14	24870	9
	268.4	-11	9241	3
	272.0	-8	33637	13
	277.9	-2	9835	4
	278.8	-1	85226	32
	279.8	0	40945	15

Supplementary Figure 1. Characterization of gene-edited cells by flow cytometry and DNA fragment length analyses.

JVM-3 cells deficient in (A) CD43 (gene: *SPN*; probe: monoclonal antibody L10), (B) CD45 (gene: *PTPRC*; probe: monoclonal antibody 2D1), (C) CD162/PSGL-1 (gene: *SELPLG*; probe: monoclonal antibody KPL1), (D) GNE (gene: *GNE*; probe: recombinant CD22/Siglec-2-Fc), (E) ST6Gal-I (gene: *ST6GAL1*; probe: recombinant CD22/Siglec-2-Fc), (F) ST6GalNAc-IV (gene: *ST6GALNAC4*; probe: MAL-II), (G) ST8Sia-IV (gene: *ST8SIA4*; probe: monoclonal antibody 2-2B), and MEC-1 cells deficient in (H) Core 2 GlcNAc transferase 1 (gene: *GCNT1*; probe: MAL-II) were characterized by flow cytometry and DNA fragment length analyses.

For flow cytometry (left), the gene-edited and control cells were stained with the antibody or lectin (indicated in parentheses on the title line), with appropriate secondary reagent if necessary, and subjected to flow cytometry. Gray histogram represents control cells stained with negative control probe (isotype-control antibody or secondary reagent alone). For DNA fragment length analysis (center: electropherograms, right: summary tables of DNA fragment lengths), genomic DNA samples extracted from the gene-edited and control cells were subjected to gene-specific PCR with a pair of primers (one FAM-labeled, the other unlabeled; Supplementary Table 3), and the fragment lengths of PCR products were analyzed by capillary electrophoresis. Unedited allele in gene-edited cells (showing the same fragment length as wild-type allele) is highlighted in red characters.

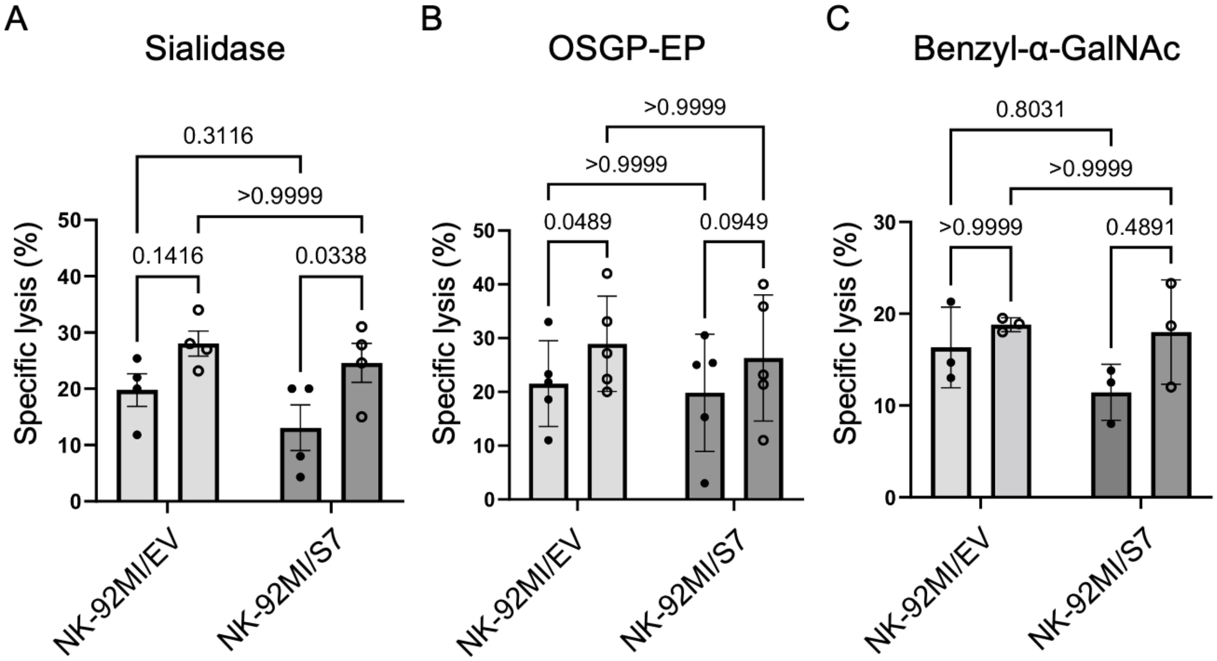

Supplementary Figure 2. Effects of sialidase, OSGP-EP, and benzyl-α-GalNAc treatment of JVM-3 cells on the NK cytotoxicity by NK-92MI/EV and NK-92MI/S7.

JVM-3 cells were treated without (solid circle) or with (open circle) (A) sialidase, (B) OSGP-EP, or (C) benzyl-α-GalNAc (72 h) and subjected to NK cytotoxicity assay using NK-92MI/EV and NK-92MI/S7 cells (E:T ratio = 2). NK-92MI/EV cells showed higher cytotoxic activity toward JVM-3 cells compared with NK-92MI/S7, but the difference was not statistically significant, owing to the large experiment-to-experiment deviation and limited number of experimental repeats. All treatments made JVM-3 cells more sensitive to cytotoxicity by both NK-92MI/EV and NK-92MI/S7 cells, and the treatment-induced enhancement of cytotoxicity appeared to be larger when NK-92MI/S7 cells were used as effector cells. However, the difference was not statistically significant in most cases, owing to the large experiment-to-experiment deviation and limited number of experimental repeats.

Cytotoxicity assays were conducted in technical triplicates, and repeated three to five times. Each dot represents an average of technical triplicates. Bars indicate SD of independent experiments. Statistical analyses were conducted by two-way ANOVA followed by Bonferroni's post-hoc test. Numbers above horizontal bars indicate adjusted *P* values.

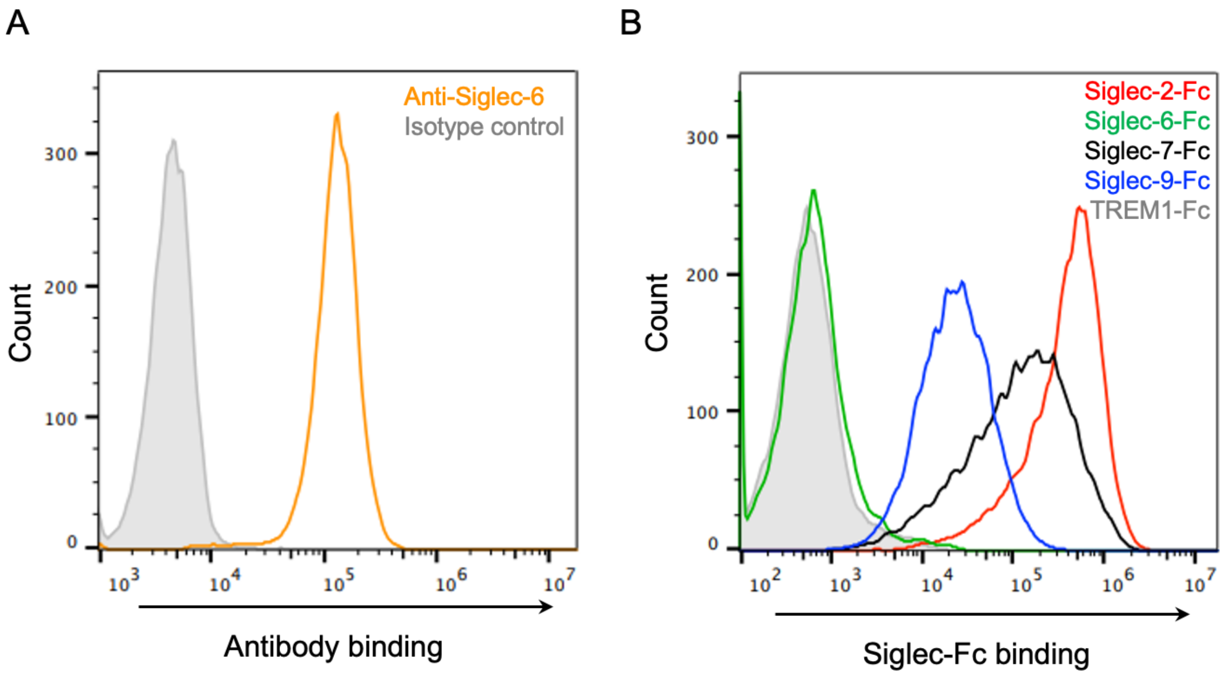

Supplementary Figure 3. Presence of Siglec-6 on NK-92MI cells and absence of Siglec-6 ligand on JVM-3 cells.

(A) NK-92MI cell line was stained with an anti-Siglec-6 antibody, followed by a PE-conjugated anti-mouse IgG (115-116-068, Jackson ImmunoResearch), and subjected to flow cytometry. Anti-Siglec-6 antibody (orange; clone 767329, R&D Systems) and isotype control antibody (gray) were used in the experiment. No other Siglec was found to be highly expressed (data not shown). (B) JVM-3 cell line was stained with recombinant Siglec–Fc. CD22/Siglec-2–Fc (red), Siglec-6–Fc (green), Siglec-7–Fc (black), Siglec-9–Fc (blue), and TREM1–Fc (gray, negative control) were used in the experiment.

Supplementary Table 1. Summary of Taiwanese patients with chronic lymphocytic leukemia.

Patient #	Age	Gender (M/F)	IGHV mutation	Siglec-2 binding (MFI)	Siglec-7 binding (MFI)	Siglec-9 binding (MFI)	GCNT1 mRNA (normalized by *PGK1*)	ST6GALNAC4 mRNA (normalized by *PGK1*)	Binet stage	Rai stage	Treatment
1	68	M	Unmutated	39698	31148	8624	9.6E-04	6.6	A	1	–
2	56	F	ND	11817	4939	438	ND	ND	B	1	–
3	66	F	ND	21561	5700	846	ND	ND	C	3	Chemo-immunotherapy
4	60	F	ND	3389	717	311	ND	ND	C	3	Chemo-immunotherapy
5	55	F	ND	11662	9172	613	ND	ND	A	1	Chemotherapy
6	50	M	ND	15090	11869	1274	ND	ND	A	3	Chemotherapy
7	51	F	ND	3565	5712	327	ND	ND	A	1	–
8	74	M	Mutated	3628	6225	247	3.6E-05	1.0	B	1	–
9	40	M	Unmutated	5139	3442	489	7.7E-02	2.2	B	2	–
10	68	F	Mutated	8529	4463	423	1.8E-03	2.0	B	2	–
11	77	F	Mutated	11208	10173	552	0	1.8	A	0	–
12	44	F	Unmutated	17109	16192	828	2.0E-03	1.4	A	1	–
13	73	M	Mutated	2148	1720	196	2.2E-02	1.7	C	4	–
14	68	F	Unmutated	13636	8816	641	1.4E-03	4.1	A	0	–
15	63	F	ND	1866	680	181	ND	ND	B	1	–
16	58	F	Mutated	10354	8090	379	2.4E-03	1.5	B	1	–
17	63	F	Mutated	9670	7262	869	8.8E-04	0.7	C	4	–

ND: not determined; –: not treated.

Supplementary Table 2. TaqMan reagents used for quantitative real-time polymerase chain reaction analyses.

Gene	Assay ID
PGK1	Hs99999906_m1
GCNT1	Hs01922706_s1
SPN	Hs01872322_s1
PTPRC	Hs04189704_m1
SELPLG	Hs04276253_m1
ST3GAL1	Hs00161688_m1
ST3GAL2	Hs00199480_m1
ST3GAL3	Hs00544033_m1
ST3GAL4	Hs00272170_m1
ST3GAL5	Hs01105377_m1
ST3GAL6	Hs01048197_m1
ST6GALNAC1	Hs00300842_m1
ST6GALNAC2	Hs01032565_m1
ST6GALNAC3	Hs00541761_m1
ST6GALNAC4	Hs00205241_m1
ST6GALNAC5	Hs00229612_m1
ST6GALNAC6	Hs00203739_m1
ST8SIA1	Hs00268157_m1
ST8SIA2	Hs00916611_m1
ST8SIA3	Hs01026908_m1
ST8SIA4	Hs00379924_m1
ST8SIA5	Hs00203298_m1
ST8SIA6	Hs02341873_m1

10

Supplementary Table 3. Sequences of sgRNA for CRISPR/Cas9 gene editing and PCR primers for DNA fragment length analyses.

Gene	SgRNA sequence	Forward primer	Reverse primer	PCR product (bp)	Annealing temperature (°C)
SPN	GGCTCGCTAGTAGAGACCAA	TCTTGCTCCTGCCTGTTTGC	AGGTTGTTGGCTCAGGTAAAGG	309	62
PTPRC	GCTTGGGTGGAAGTATTGTC	GCAAAGATGCCCAGTGTTCC	TCCACTCCCTAACAACATGC	386	62
SELPLG	GGCCCAGTAGGATCAGCAAC	CCAAGGGTGAAACTGTCTTG	TCTCCATAGCTGCTGAATCC	415	61
ST6GAL1	GCCCCAGACTCTTTAACACC	GAAAAAGTTCAGCTGCTGCG	TTTGCAGCCTAGGGATAAGG	311	59
ST6GALNAC4	GCTCATCATCCTGTGCTCCG	GCTTGGCATCCCCAGGTAAG	ACTCACCTTCCCATCTGGCA	364	63
ST8SIA4	GACCAGGAGACGCAACTCAT	GCTCTCCTCGCATTTTACAG	CTAACCATCACTCTACCCTC	311	56
GCNT1	GGCTGAGGACGTTGCTGCGA	CATTTCAAGATGCCGTTGCAG	CAGCAAGCTCCAAGTGTCTGA	281	65
GNE	GTTAATGCCAAACATGATCG	TCACACATAAGTGGAGGTGC	TCCTTCTAGCACACTGTTGC	412	59
Control	GCGAGGTATTCGGCTCCGCG	–	–	–	–

Supplementary Dataset 1. Proteins identified as Siglec-7 counterreceptor candidates by proximity labeling and mass spectrometry. (Please see separate Excel file)

12
